# Prussian Blue Nanoparticles Confined in Chitosan for In Vivo Cesium Ion Removal

**DOI:** 10.3390/nano16090544

**Published:** 2026-04-29

**Authors:** Irina E. Bordianu-Antochi, Afitz Da Silva, Giovanni Massasso, Françoise Quignard, Vanja Stojanovic, Magali Gary-Bobo, Joulia Larionova, Yannick Guari

**Affiliations:** 1 ICGM, University of Montpellier, CNRS, ENSCM, 34293 Montpellier, France; irina-elena.antochi@chu-lyon.fr (I.E.B.-A.); giovanni.massasso@saint-gobain.com (G.M.); joulia.larionova@umontpellier.fr (J.L.); 2 IC, Hospices Civils de Lyon (HCL), 69495 Lyon, France; 3 IBMM, University of Montpellier, CNRS, ENSCM, 34293 Montpellier, France; afitz.da.silva.hsj@ssss.gouv.qc.ca (A.D.S.); vanja.stojanovic@pmf.edu.rs (V.S.); magali.gary-bobo@umontpellier.fr (M.G.-B.); 4 CHU Sainte-Justine Research Centre, University of Montreal, Montreal, QC H3T 1C5, Canada; 5 Saint Gobain Research Provence, 84306 Cavaillon, France; 6 Faculty of Sciences and Mathematics, University of Nis, 18000 Nis, Serbia

**Keywords:** cesium decorporation, Prussian blue nanoparticles, chitosan nanocomposite, in vivo detoxification

## Abstract

The development of efficient and biocompatible sorbent nanomaterials for cesium removal is critical for environmental and biomedical decontamination. Here, hybrid composites based on ultra-small Prussian blue or Zn Prussian blue-type nanoparticles confined within porous chitosan beads are proposed for Cs^+^ extraction. Nanoparticle confinement ensures homogeneous dispersion and improved accessibility of ion-exchange sites, while preserving the porous polymeric network, as confirmed by physicochemical characterization. Cs^+^ adsorption was investigated under neutral and acidic conditions (pH 7.2 and 1.2), at concentrations of 0–9 mmol/L and contact times of 0–50 h, showing efficient uptake and favorable kinetics, with confirmed stability in simulated gastric fluid. In vivo performance was assessed in a mouse model of cesium contamination (70 mg Cs^+^/kg). Treatment with nanocomposites (225 mg/kg) was compared to bulk Prussian blue (75 mg/kg), revealing enhanced detoxification efficiency. Histological analysis of liver, spleen, and kidney tissues showed no detectable structural damage, consistent with unchanged systemic biomarkers. Overall, the proposed chitosan-confined Prussian blue-type nanocomposites combine high Cs^+^ removal efficiency, kinetic accessibility, and in vivo safety, highlighting their potential for decorporation applications.

## 1. Introduction

Contamination of living organisms by monovalent radioactive cations, such as cesium (Cs^+^), is a serious problem that concerns populations living around nuclear power plants, laboratories, or industrial sites, as well as hospitals using clinical applications of radioactivity. Radioactive cesium isotopes are among the most abundant fission products of uranium and are hazardous to human health. Among them, ^134^Cs and ^137^Cs are gamma-emitting radionuclides with half-lives of approximately 2 and 30 years, respectively, and are mainly present in fission products. Radiation accidents, such as those that occurred in Goiânia (Brazil), Chernobyl (Ukraine), and more recently Fukushima (Japan), caused the release of these isotopes into the atmosphere, their accumulation in the food chain, and their persistence in the environment over long periods. Accidental exposure can induce internal contamination of humans and animals through the unintended absorption of Cs^+^ via various routes, including oral, respiratory, and percutaneous pathways [1,2]. In addition to its environmental persistence, internal exposure to radioactive Cs^+^ is associated with significant health risks, including increased cancer incidence due to prolonged gamma irradiation. Chronic exposure may also lead to cardiovascular and metabolic disorders, particularly in vulnerable populations. Environmental contamination pathways include deposition on soils and vegetation, followed by transfer through the food chain via agricultural products, milk, and meat consumption, which constitute the primary sources of long-term human exposure [3,4,5,6]. These biological effects are closely related to the ability of Cs^+^ to mimic potassium in physiological systems. Since Cs^+^ is a potassium analog, it may be transported into cells through physiological ion transport pathways, including the Na^+^/K^+^-ATPase pathway, and can be distributed particularly in soft tissues and muscles [7,8]. For this reason, the health impact of Cs^+^ contamination strongly depends on both the dose and biological residence time [9].

To date, only the so-called insoluble form of Prussian blue, marketed as Radiogardase^®^, is known as the FDA-approved treatment for Cs^+^ ion decontamination [1,10,11,12,13,14]. Prussian blue is a bulk cyano-bridged iron-based coordination polymer with the chemical formula Fe_4_[Fe(CN)_6_]_3_·13H_2_O, which shows high affinity for several monovalent cations of similar ionic radius over a wide pH range due to selective insertion into the tetrahedral sites of its three-dimensional face-centered cubic network [15]. After oral ingestion, bulk Prussian blue is not absorbed through the gastrointestinal wall and remains in the gastrointestinal tract for several hours. It captures monovalent cations excreted via bile by the liver and reduces their enterohepatic circulation, thereby decreasing the biological half-life by approximately 69% for adults. Beyond Prussian blue, several conventional approaches have been explored for cesium removal, particularly in environmental remediation, including adsorption using zeolites, ion-exchange resins, and clay minerals, as well as membrane filtration and solvent extraction techniques [16,17,18,19,20]. While these methods can exhibit high efficiency in aqueous systems, their application to in vivo decorporation remains limited due to issues such as poor selectivity in complex biological media, slow kinetics, a lack of biocompatibility, and the inability to prevent reabsorption within the gastrointestinal tract. Consequently, Prussian blue remains the only clinically approved option for Cs^+^ decorporation, despite its known limitations. Indeed, treatment efficiency is still limited for adolescents (46%) and young children (43%) [21].

Therefore, the development of new biomaterials for efficient and selective in vivo Cs^+^ decontamination is highly desirable. In this line of thought, it has been demonstrated that nanocomposites containing nanoparticles of Prussian blue [22,23,24] or its analogs [25,26,27,28,29,30,31] show improved adsorption kinetics and cesium uptake capacity compared to the corresponding bulk materials. Environmental and biomedical cesium decontamination is a highly topical research area that has been the subject of numerous reviews, including very recent ones [32,33,34]. Among the composites investigated, those based on a biopolymer combined with Prussian blue or its analogs play an important role in remediation strategies. However, despite the growing interest in such materials, the demonstration of composite efficiency for cesium decorporation remains limited, with only a few reported examples. To date, only three studies have described the successful use of Prussian blue nanoparticles and their nanocomposites for in vivo Cs^+^ decontamination, including cellulose- [35], silica- [36], or carbon sponge [37]-based nanocomposites, as well as Prussian blue confined in the AONYS^®^ reverse micellar system [38].

In this article, we describe new nanocomposite materials for highly efficient in vivo Cs^+^ decontamination based on Prussian blue-type nanoparticles embedded in porous chitosan beads. The chitosan beads were chosen as the matrix because chitosan is a non-toxic, biocompatible, biodegradable, and bioactive polymer derived from partial deacetylation of chitin, and is approved for use in humans [39,40]. In addition, chitosan contains free amino groups capable of coordinating metal ions, thereby providing sites for nanoparticle anchoring. Chitosan also exhibits good water affinity, and when shaped into beads and dried under supercritical CO_2_ conditions, it can develop a porous structure [41]. Two types of nanoparticles, Prussian blue and its Zn^2+^-based analog, were synthesized and covalently anchored within the chitosan beads. The Zn^2+^-based system was also investigated due to the low toxicity and biological relevance of zinc, a trace essential element in physiological systems [42]. The adsorption capacity of the nanocomposites was investigated in water and in simulated gastric fluid. Particular emphasis was placed on the in vivo Cs^+^ decontamination properties and biocompatibility assessment of the nanocomposites. Compared to conventional bulk materials and existing treatment strategies, the proposed nanocomposite system offers several advantages, including enhanced surface area, improved adsorption kinetics, and better accessibility of active sites due to nanoparticle dispersion within a porous and biocompatible matrix [43]. Moreover, the use of chitosan enables safe gastrointestinal transit while potentially increasing interaction time with Cs^+^ ions, thereby improving decorporation efficiency.

## 2. Materials and Methods

All of the chemical reagents used in these experiments were analytical grade: Fe(BF_4_)_2_·6H_2_O, chitosan and acetic anhydride (C_4_H_6_O_3_) were supplied by Sigma-Aldrich (St. Louis, MO, USA); Zn(NO_3_)_2_·6H_2_O and CsCl by Thermo Scientific Chemicals (Haverhill, MA, USA); ethanol (96%, *w*/*w*) was technical grade and was supplied by Sodipro (Echirolles, France).

### 2.1. AI Tool Usage

**Figure 1:**
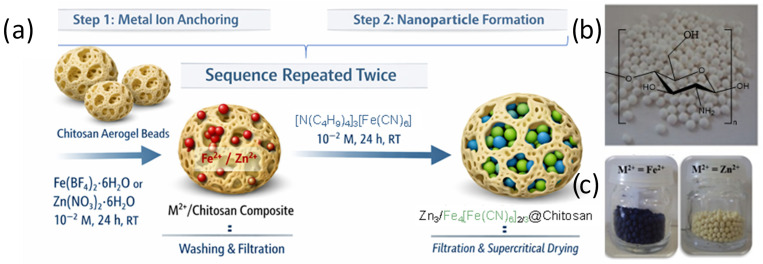
(**a**) Schematic representation of the synthesis of the nanocomposites Fe_4_[Fe(CN)_6_]_3_/chitosan (**1**) and Zn_3_[Fe(CN)_6_]_2_/chitosan (**2**). Photographs of (**b**) pristine chitosan beads and (**c**) nanocomposites **1** (left) and **2** (right).

ChatGPT (GPT-5, March 2026 version) was used to generate a first draft of Figure 1.

### 2.2. Synthesis

The typical synthesis of the M^2+^/chitosan-based nanocomposites (M = Fe^2+^ or Zn^2+^) is based on a stepwise strategy involving sequential coordination of metal precursors and the growth of Prussian blue-type nanoparticles within a chitosan matrix (Figure 1a). This approach has previously been employed for the preparation of nanocomposites containing other Prussian blue analog nanoparticles [44,45,46,47,48]. In the first step, porous chitosan aerogel beads [49] (50 mg) are immersed for 24 h at room temperature in a 10^−2^ M methanolic solution of Fe(BF_4_)_2_·6H_2_O (or [Fe(H_2_O)_6_]^2+^) or in a 10^−2^ M acetonitrile solution of Zn(NO_3_)_2_·6H_2_O (or [Zn(H_2_O)_6_]^2+^), allowing coordination of the divalent metal ions to the –NH_2_ functional groups of pristine chitosan beads (Figure 1b). The resulting M^2+^/chitosan beads are then washed twice with the corresponding solvent. In the second step, nanoparticle growth is achieved by adding the M^2+^/chitosan composite (50 mg) to a 10^−2^ M solution of [N(C_4_H_9_)_4_]_3_[Fe(CN)_6_] in methanol (for Fe^2+^/chitosan) or acetonitrile (for Zn^2+^/chitosan), followed by stirring for 24 h at room temperature. The beads are then filtered and washed twice with methanol. Subsequently, they are successively re-treated with the appropriate divalent metal ion solution and [Fe(CN)_6_]^3−^ precursor, and this sequential M^2+^/[Fe(CN)_6_]^3−^ treatment is repeated twice to increase nanoparticle loading within the porous chitosan matrix. Finally, the beads are dried under supercritical CO_2_ conditions to yield aerogel nanocomposites, namely Fe_4_[Fe(CN)_6_]_3_/chitosan (**1**) and Zn_3_[Fe(CN)_6_]_2_/chitosan (**2**), while preserving the original textural properties of the chitosan framework. Notably, the color of the materials reflects that of the corresponding bulk phases: pristine chitosan beads are white, nanocomposite **1** appears deep blue, and nanocomposite **2** pale yellow (Figure 1c).

Elemental analysis for **1** (wt%): C, 35.05; H, 5.56; N, 14.98; Fe, 15.09. The determined formula is Fe_4_[Fe(CN)_6_]_3_@(chitosan)_10.8_; IR (cm^−1^): ν(OH) 3405; ν(CH) 2940, 2875; ν(CN) (Fe^2+^–NC–Fe^3+^) 2078; ν(CO) and δ(NH_2_) 16 51; δ(NH) 1558; δ(CH) 1385; ν(CO) 1065; ν(C–O–C) 1144, 881.

Elemental analysis for **2** (wt%): C, 37.06; H, 5.07; N, 15.34; Fe, 7.08; Zn, 12.32. The determined formula is Zn_3_[Fe(CN)_6_]_2_@(chitosan)_6_; IR (cm^−1^): ν(OH) 3395; ν(CH) 2 932, 2880; ν(CN) 2173, 2100; ν(CO) and δ(NH_2_) 1645; δ(NH) 1549; δ(CH) 1379; ν(CO) 1059; ν(C–O–C) 1146, 879.

[N(C_4_H_9_)_4_]_3_[Fe(CN)_6_] was prepared according to the literature procedures [50]. The porous chitosan beads (aerogel) were synthesized as previously described [45,46,47]. The degree of acetylation of chitosan of 10% (which is the percent of remaining acetyl groups) was measured by IR spectroscopy.

### 2.3. Physical Measurements

Specific surface areas were determined by the Brunauer–Emmett–Teller (BET) method with a Micromeritics ASAP 2010 analyzer (Malvern, UK). Samples were outgassed under vacuum at 60 °C over night prior to analysis. Surface area was determined using BET method. The specific surface area decrease was used as a proxy for pore occupation by the incorporated phase. Assuming that the reduction in surface area is proportional to pore filling, the following relation was applied:

(1) $$Pore\;filling=\frac{S_{0}-S}{S_{0}}$$

where *S*_0_ is the initial specific surface area of the pristine material and *S* is the specific surface area measured after modification. Under this assumption, any decrease in surface area is interpreted as progressive occupation of the porous network, allowing a relative comparison of the extent of pore filling between samples. Elemental analyses were performed by the Service Central d’Analyse (CNRS, Vernaison, France). Nitrogen and carbon content in the samples was determined using LECO instrument (St. Joseph, MI, USA). The samples were heated at 3000 °C under oxygen. Nitrogen and carbon were transformed respectively in NOx and CO_2_ and detected by using an IR detector. Cesium species concentrations in solution for sorption experiment in inactive conditions were measured using Metrohm ionic chromatography (Herisau, Switzerland). Solutions were injected in a column as mobile phase. Cs species were retained on the stationary phase and then eluted after 20 min and analyzed by conductivity. IR spectra were recorded on a Perkin Elmer 1600 spectrometer with a 4 cm^−1^ resolution (Waltham, MA, USA). Transmission Electron Microscopy (TEM) observations were carried out at 100 kV using a JEOL 1200 EXII (Tokyo, Japan). Samples for TEM measurements were prepared using ultramicrotomy techniques or by dissolution of the chitosan matrix in acidic solutions for the nanocomposite chitosan beads. Ultramicrotomy technique consists of suspending the material in a resin which is polymerized at low temperature (i.e., 70 °C), then slices of circa 60 to 100 nm were cut with an ultramicrotome apparatus LEICA UC 7 equipped with a diamond knife (Wetzlar, Germany). This technique allows visualizing both the nanoparticles and the chitosan matrix. In the case of the colloids, a drop of the solution was deposited on a copper grid. EDS analyses were performed by using an Environmental Secondary Electron Microscope FEI Quanta 200 FEG (Hillsboro, OR, USA) coupled with an Electrons Dispersive Spectrometer Oxford INCA detector (Abingdon-on-Thames, UK). Powder X-ray diffraction patterns were measured on a Bruker D8 advanced Diffractometer (Billerica, MA, USA) in Bragg–Brentano geometry using Ni-filtered Cu-Kα radiation. The measurement parameters are: step size, 0.02 (2q); counting time, 15 s per step.

### 2.4. Sorption Kinetics

A total of 40 mL of 0.1 mmol·L^−1^ CsCl in aqueous solution was put in contact with 20 mg of materials and experiments were conducted from 30 min to 48 h with shaking. After reaching the contact time, solid components were separated from the liquid phases and the remaining cesium concentration of the supernatant was measured by using ionic chromatography. The adsorbed cesium quantity (*Q_t_* in mmol·L^−1^) at time *t* is expressed as follows:

(2) $$Q_{t}=(C_{0}-C_{t})\frac{V}{m}$$

where *C*_0_ (mmol·L^−1^) is the initial concentration of cesium, *C_t_* (mmol·L^−1^) is the remaining concentration of cesium in solution after the specified time t (min), *V* (L) is the volume of the solution and *m* (g) is the mass of solid used.

### 2.5. Sorption Isotherms

For adsorption isotherm studies, a first parent solution of 14 mmol·L^−1^ of Cs^+^ was prepared by dissolving CsCl in deionized water. Solutions with Cs^+^ concentrations ranging from 0.1 to 14 mmol·L^−1^ were prepared by diluting the parent solution with deionized water. For the isotherm experiments, 20 mg of sorbent nanocomposites (or bulk Prussian blue) were added to 40 mL of cesium chloride solution and stirred for 24 h, the time at which the equilibrium was reached. After 24 h the solutions were filtered and the remaining cesium concentration was determined using ionic chromatography. To establish the adsorption isotherm, the remaining solute concentration of the cesium at equilibrium, *C_e_* (mmol·L^−1^), was compared with the concentration of the cesium retained on solid particles (nanocomposite or bulk materials), *Q_e_* (mmol·g^−1^). The relationship *Q* = *f*(*C*) is known as a “sorption isotherm”. The cesium concentration retained in the solid particles at equilibrium is given by the equation:

(3) $$Q_{e}=\left(C_{i}-C_{e}\right)\frac{V}{m}$$

where *V* is the volume of solution (L), *m* is the mass of the sorbent (nanocomposite or bulk materials) used (g), *C_i_* is the initial concentration of cesium in solution (mmol·L^−1^) and *C_e_* is the equilibrium concentration of the cesium in solution (mmol·L^−1^).

### 2.6. Isotherm in Simulated Gastric Fluid

The simulated gastric fluid was prepared by mixing 0.035 mol·L^−1^ NaCl, 0.084 mol·L^−1^ HCl and 9.14 × 10^−5^ mol·L^−1^ of pepsin. The isotherm experiments in simulated gastric fluid have been performed in the same manner as in water with the difference that at the end of the experiment the residual solid was separated from the remaining Cs^+^ containing solutions by centrifugation at 20,000 rpm for 10 min and the remaining cesium concentration was then determined using ionic chromatography.

### 2.7. In Vivo Studies

Animal experiments were approved and conducted in accordance with local and national authorities for the care and use of laboratory animals. The accreditation number of the laboratory for the experiments conducted in this work is B34-172-25. Twenty-four-month-old female mice C57BL/6 were purchased from Harlan (Indianapolis, IN, USA), and used after acclimatization for a week. During acclimatization and experiment periods, animals were given free access to food pellets and tap water. Animals were housed at 22 °C in a 12 h light/12 h dark cycle. Before the beginning of the experimentation, mice were divided into 5 groups of 4 mice. All mice were subcutaneously injected with physiological serum supplemented (groups 2, 3, 4 and 5) or not (group 1) with cesium (70 mg/kg). For the data analysis and graphical representation, group 1 is called “Control”, group 2 is “Cs”, group 3 is “Cs + PB”, group 4 is “Cs + **1**” and group 5 is “Cs + **2**”. It means that just after cesium injection, groups 3, 4 and 5 were respectively treated per os (using feeding needles: 20 G, 1 po) with 75 mg/kg of Prussian blue, or 225 mg/kg of (**1**) or 225 mg/kg of (**2**). Group 2 did not receive treatment after cesium injection. Stools were collected during the 2 h following treatments to measure, by inductively coupled plasma mass spectrometry from Agilent Technologies (Santa Clara, CA, USA), the quantity of cesium eliminated under different conditions. The mice were sacrificed 48 h after treatments. Urine and blood were collected to determine the levels of inflammatory biomarkers such as TNFα, creatinine and alanine aminotransferase (ALT). Serum was separated from blood by centrifugation (10 min, 4000 rpm). Creatinine activity was determined in urine and serum by Jaffé method. Expression of TNFα was quantified in serum by specific ELISA immunoassay and expression of circulating ALT was quantified by enzymatic assay. Organs involved in metabolism (livers, spleen and kidneys) were collected and hematoxylin/eosin-stained sections from paraffin-embedded tissues were macroscopically examined.

## 3. Results

### 3.1. Synthesis and Characterization of M_x_[Fe(CN)_6_]_y_/Chitosan Nanocomposite Beads (M = Fe^3+^, x = 4, y = 3 or M = Zn^2+^, x = 3, y = 2)

The detailed synthesis of the composites is described in Section 2, Materials and Methods. The nanocomposites were further characterized by IR spectroscopy, particularly in the spectral region 2000–2300 cm^−1^ corresponding to the CN^−^ stretching mode, which is a fingerprint of the structural and electronic environment in Prussian blue and its Zn^2+^ analog. The IR spectrum of nanocomposite **1** displays the characteristic band at 2078 cm^−1^ observed for bulk Prussian blue, whereas nanocomposite **2** shows two bands at 2173 and 2100 cm^−1^, also consistent with those reported for the bulk analog (Table 1, Figure S1) [50,51]. In addition, the spectra retain the characteristic vibrational bands of the chitosan matrix. Elemental analysis indicates nanoparticle loadings of approximately 33 and 39 wt% for nanocomposites **1** and **2**, respectively. The specific surface area of the nanocomposite beads were investigated by nitrogen adsorption–desorption measurements. In both cases, the isotherms exhibit a pronounced hysteresis loop typical of a type IV curve, which is characteristic of mesoporous materials according to the IUPAC classification (Figure S2). The specific surface area decreased to 329 and 270 m^2^·g^−1^ for both nanocomposites **1** and **2**, respectively, compared with 382 m^2^·g^−1^ for pristine chitosan beads. Assuming a uniform filling of the pores, the surface covered by the nanoparticles becomes inaccessible to nitrogen adsorption (BET measurement), leading to a proportional decrease in the accessible surface area (Table 1). This decrease is thus consistent with pore filling by the nanoparticles, estimated at 14% and 29% for nanocomposites **1** and **2**, respectively.

**Figure 2:**
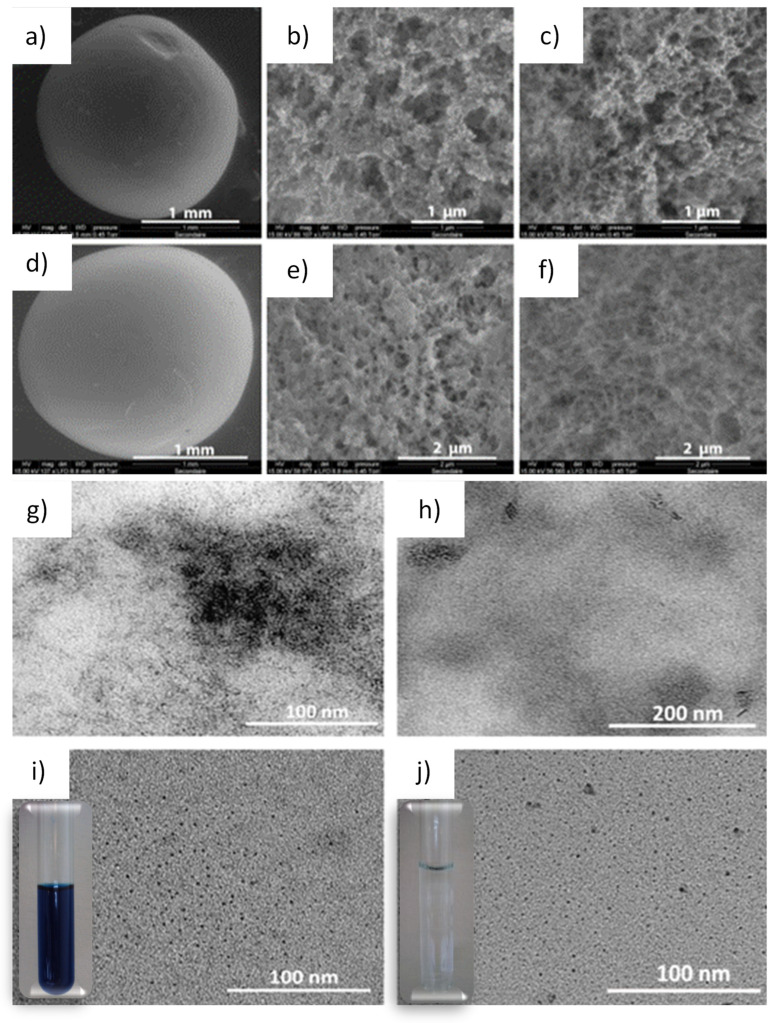
SEM images of nanocomposites **1** and **2**: (**a**,**d**) whole beads; (**b**,**e**) external surfaces; (**c**,**f**) internal surfaces. TEM images of **1** (**g**,**i**) and **2** (**h**,**j**) in the chitosan matrix and after dissolution in aqueous solution (pH 1.2). Insets: photographs of the nanoparticle suspensions obtained after dissolution of the chitosan matrix.

To gain insight into the morphological characteristics of the developed nanocomposites, scanning electron microscopy (SEM) analyses were performed on both the external and internal surfaces of the beads after cleavage. Figure 2 presents SEM images of whole beads (Figure 2a,d), their outer surfaces (Figure 2b,e), and their inner surfaces after cleavage (Figure 2c,f). The internal porous structure of the pristine chitosan beads is preserved after the formation of Prussian blue-type nanoparticles within the pores. No evidence of bulk Prussian blue or its analog is observed on the bead surfaces. Transmission electron microscopy (TEM) observations were carried out on the nanocomposites both within the chitosan matrix and after dissolution of the chitosan in an acidic aqueous solution (pH 1.2). In both cases, ultra-small nanoparticles with sizes ranging from 1 to 3 nm are observed, homogeneously dispersed either within the chitosan matrix (Figure 2g,h and Figures S3 and S4) or in the acidic aqueous solution (Figure 2i,j). The nanoparticle suspensions obtained after matrix dissolution remain perfectly stable for several months, with no precipitation observed over time (insets of Figure 2i,j).

### 3.2. Cesium Capture Studies

**Figure 3:**
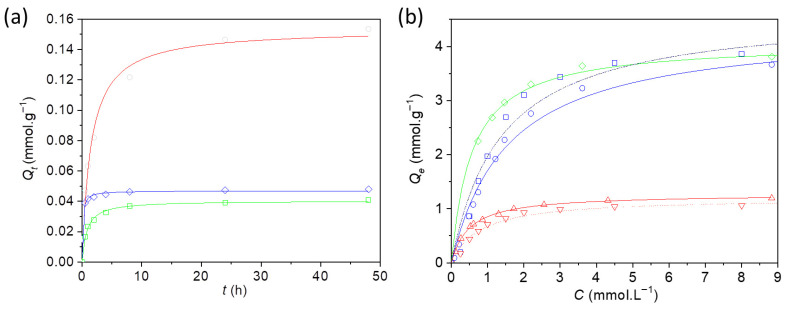
(**a**) Kinetics of Cs^+^ sorption experiments performed for nanocomposites **1** (-◇-) and **2** (-□-) and for the bulk Prussian blue (-○-). Solid lines represent the fit of the experimental points with the pseudo-second order model (Equation (4)). (**b**) Isotherms for Cs^+^ absorption performed for **1** (-○-), **2** (-◇-) and bulk Prussian blue (-△-) in water and for **1** (^..^□^..^) and bulk Prussian blue (^..^▽^..^) in simulated gastric fluid (pH = 1.2). Solid lines represent the fit with the Langmuir model (Equation (5)).

Cs^+^ sorption experiments were conducted for nanocomposites **1** and **2**, as well as for bulk Prussian blue for comparison in water at neutral pH. Sorption kinetics were first investigated to determine the time required to reach equilibrium. Aqueous solutions of cesium chloride were contacted with the sorbents for contact times ranging from 30 min to 48 h. After the designated time, the sorbents were separated, and the residual Cs^+^ concentration in the supernatant was measured. Kinetic data were fitted using a pseudo-second-order model [52], where *Q_t_* (mmol·g^−1^) is the amount of Cs^+^ entrapped at time *t* (min), *Q_e_* is the amount at equilibrium, and *k* (g·mmol^−1^·min^−1^) is the kinetic constant (Equation (4), Figure 3a).

(4) $$Q_{t}=\frac{kQ_{e}^{2}t}{1+kQ_{e}^{2}t}$$

The kinetic curves indicate that Cs^+^ sorption is significantly faster for the nanocomposites, reaching equilibrium after approximately 8 h, whereas bulk Prussian blue requires nearly 24 h (Figure 3a). This observation is consistent with previous reports showing that Cs^+^ sorption by bulk Prussian blue or alkali-free Prussian blue analogs (e.g., Cu^2+^, Co^2+^, Ni^2+^) is very slow [53].

To fully characterize Cs^+^ uptake, sorption isotherms were obtained at increasing Cs^+^ concentrations. The isotherms were fitted using the Langmuir model [54], where *Q_e_* (mmol·g^−1^) is the amount of Cs^+^ adsorbed at equilibrium concentration *C* (mol·L^−1^), *Q_max_* (mmol·g^−1^) is the maximum adsorption capacity, and *K* (L·mol^−1^) is the affinity (Equation (5), Figure 3b). All isotherms display a concave shape with a steep increase at low concentrations, indicating a high affinity for Cs^+^ in all cases.

(5) $$\frac{Q_{e}}{Q_{max}}=\frac{KC}{1+KC}$$

Both nanocomposites efficiently capture Cs^+^, with maximum sorption capacities of 3.7 and 3.8 mmol·g^−1^ for **1** and **2**, respectively, while bulk Prussian blue shows a lower capacity of 1.2 mmol·g^−1^ (Table 1). These values are consistent with previously reported Prussian blue nanocomposites [35,36,37]. Considering that nanocomposites **1** and **2** contain 33 and 39 wt% of nanoparticles, respectively, the Cs^+^ sorption capacity normalized per gram of nanoparticles reaches 7.6 and 10 mmol·g^−1^, representing a 6–8-fold increase in efficiency. This enhanced performance is attributed to the higher accessible surface area of the nanoparticles [29]. Notably, sorption experiments performed in simulated gastric fluid (pH = 1.2) for nanocomposite **1** or bulk Prussian blue yielded results comparable to those in water at neutral pH with maximum sorption capacities of 3.9 and 1.1 mmol·g^−1^, respectively.

### 3.3. In Vivo Cesium Decontamination Potential of the Nanocomposites

The Cs^+^ decontamination potential of the nanocomposites was evaluated in vivo in mice and compared with the conventional treatment with bulk Prussian blue and the natural Cs^+^ excretion observed in untreated animals. For this purpose, a CsCl solution (70 mg·kg^−1^) was intravenously injected into mice, which were subsequently treated orally with 225 mg·kg^−1^ of nanocomposites or with an equivalent dose of bulk Prussian blue (75 mg·kg^−1^). Since previous studies have shown that the use of nanoparticles may induce a shift in the excretion pathway toward preferential fecal elimination [38], Cs^+^ excretion was monitored through the analysis of collected feces and compared with that of untreated mice.

**Figure 4:**
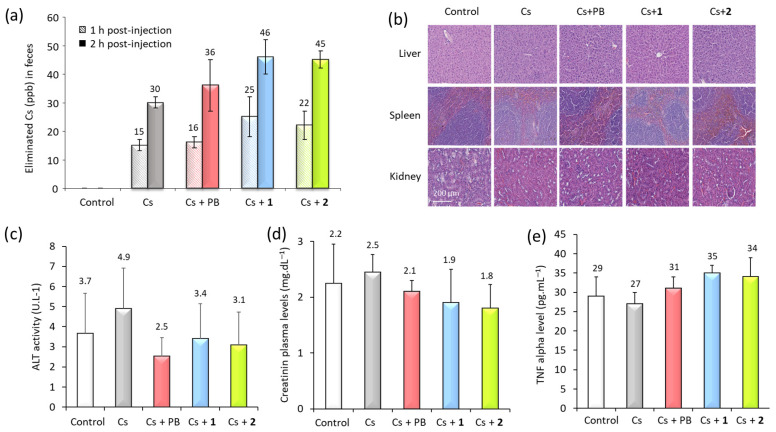
(**a**) Cesium elimination after treatment with nanocomposites **1** and **2** or bulk Prussian blue (PB), compared with untreated mice and mice without Cs^+^ injection used as controls. Cs^+^ levels in stools were quantified by ICP-MS in samples collected 1 (left bars) and 2 h (right bars) after Cs^+^ injection and treatment. (**b**) Histological analysis of organs of interest (liver, spleen, and kidney). Hematoxylin- and eosin-stained sections of paraffin-embedded tissues from control and treated mice. Plasma levels of biomarkers of the (**c**) liver (ALT), (**d**) renal (creatinine) or (**e**) systemic inflammation (TNFα). Values are means ± standard deviation.

The results presented in Figure 4a show that in untreated mice, approximately 15 ppb of Cs^+^ was excreted in feces 1 h after injection, increasing to about 30 ppb after 2 h, corresponding to the natural elimination kinetics of cesium. Under treatment with bulk Prussian blue, no significant improvement in Cs^+^ elimination was observed within this time frame, suggesting that a longer period may be required to detect a measurable effect as already observed [38]. In contrast, 2 h after treatment with the nanocomposites, the Cs^+^ concentration in the feces was approximately 50% higher than in untreated mice. These results demonstrate that the nanocomposites accelerate Cs^+^ excretion via the fecal pathway and are more effective than bulk Prussian blue under the tested conditions.

### 3.4. Biocompatibility and Safety of the Nanocomposites

The safety of the nanocomposites for cesium detoxification was also evaluated in vivo. Two days after treatment with the nanocomposites or bulk Prussian blue, mice were sacrificed and their urine, blood, and major metabolic organs (liver, spleen, and kidneys) were collected for analysis. Histological examinations revealed no apparent abnormalities or lesions in the liver, spleen, or kidneys (Figure 4b), indicating that the structural integrity of these organs was preserved. This observation was further supported by the absence of significant changes in conventional biomarkers associated with tissue function and/or systemic inflammation, including ALT (liver function), creatinine (renal function), and TNFα (systemic toxicity) (Figure 4c,d).

## 4. Conclusions

Currently, the only approved treatment for cesium decontamination is commercial Radiogardase^®^ (bulk Prussian blue). While essential in the event of a nuclear accident, its efficacy remains limited. To address this, we developed biocompatible chitosan-based nanocomposites containing Prussian blue-type nanoparticles, which show potential for cesium decontamination in vivo. These nanocomposites were found to be approximately four times more efficient than bulk Prussian blue in water or simulated gastric fluid for cesium capture. Moreover, in vivo studies demonstrated that they significantly accelerate cesium excretion via feces, with a 50% increase compared to untreated mice after 2 h of treatment. Preliminary toxicology assessments in mice further confirmed that the nanocomposites are fully biocompatible. Overall, the nanocomposites described here represent promising candidates for therapeutic applications, particularly for the decorporation of monovalent radioactive ions in the event of nuclear contamination.

## Figures and Tables

**Table 1:** Relevant characteristics of nanocomposites **1** and **2**, and bulk PB.

Sample	M^2+^/Fe Ratio	ν(CN) ^a^ cm^−1^	S_BET_ ^b^ m^2^·g^−1^	Pore Filling %	*t*_eq_ ^c^ h	*Q*_max_ ^d^ pH 7.2 mmol·g^−1^	*Q*_max_ ^d^ pH 1.2 mmol·g^−1^
**1**	-	2078	329	14	4	3.7	3.9
**2**	1.5	2100, 2173	270	29	4	3.8	-
**Bulk PB**	-	2080	-	-	20	1.2	1.1

**^a^** ν(CN): stretching vibration frequency of the C≡N bond (IR spectroscopy); **^b^** S_BET_: specific surface area determined by the Brunauer–Emmett–Teller (BET) method; **^c^** *t_eq_*: equilibrium time (time required to reach adsorption equilibrium); **^d^** *Q_max_*: maximum adsorption capacity (maximum amount adsorbed at saturation).

## Data Availability

The original contributions presented in this study are included in the article/Supplementary Materials. Further inquiries can be directed to the corresponding author.

## Supplementary Materials

The following supporting information can be downloaded at: https://www.mdpi.com/article/10.3390/nano16090544/s1, Figure S1. Infrared spectra for (a) Fe_4_[Fe(CN)_6_]_3_/chitosan (**1**) and (b) Zn_3_[Fe(CN)_6_]_2_/chitosan (**2**). Figure S2. Nitrogen adsorption-desorption isotherms for pristine chitosan beads (-●-), Fe_4_[Fe(CN)_6_]_3_/chitosan (**1**) (-■-) and Zn_3_[Fe(CN)_6_]_2_/chitosan (**2**) (-▲-). Figure S3. Size distributions of the nanoparticle size for (a) Fe_4_[Fe(CN)_6_]_3_/chitosan (**1**) and (b) Zn_3_[Fe(CN)_6_]_2_/chitosan (**2**). Figure S4. EDS elemental mapping for (a) Fe_4_[Fe(CN)_6_]_3_/chitosan (**1**) and (b) Zn_3_[Fe(CN)_6_]_2_/chitosan (**2**).
