# Supplementary material for: Prussian Blue Nanoparticles Confined in Chitosan for In Vivo Cesium Ion Removal
Source: Nanomaterials (Basel). 2026 Apr 29;16(9):544. doi: 10.3390/nano16090544 (PMC13164682; doi:10.3390/nano16090544)
Supplement: Supplementary file 1 [file nanomaterials-16-00544-s001.zip › nanomaterials-4274502-supplementary.pdf]

Electronic Supporting Information

Prussian Blue Nanoparticles Confined in Chitosan for In Vivo Cesium Ion Removal

Irina E. Bordianu-Antochi [§], Afitz Da Silva [§], Giovanni Massasso, Françoise Quignard[†], Vanja Stojanovic, Magali Gary-Bobo, Joulia Larionova and Yannick Guari

a)

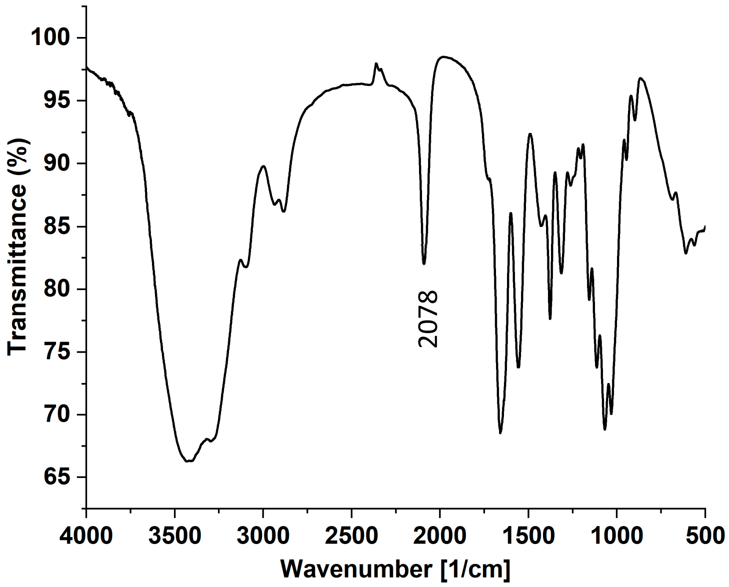

b)

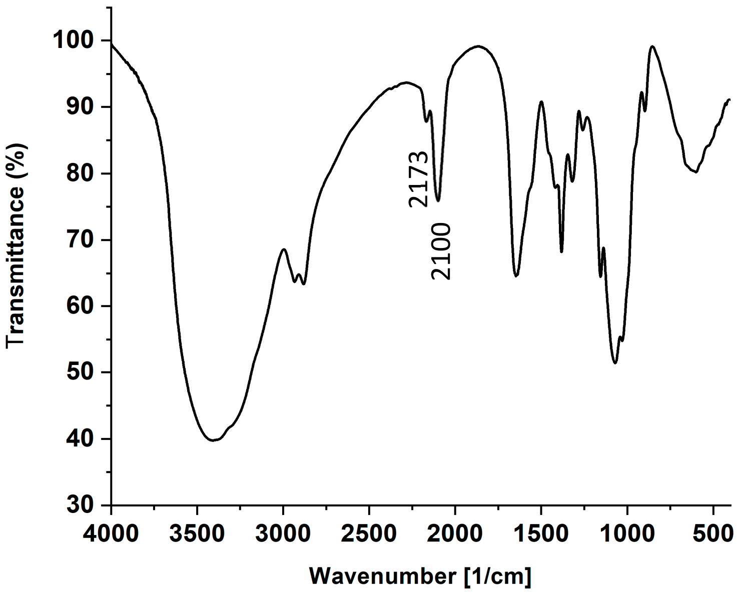

Figure S1. Infrared spectra for (a) $Fe_4[Fe(CN)_6]_3$/chitosan (**1**) and (c) $Zn_3[Fe(CN)_6]_2$/chitosan (**2**).

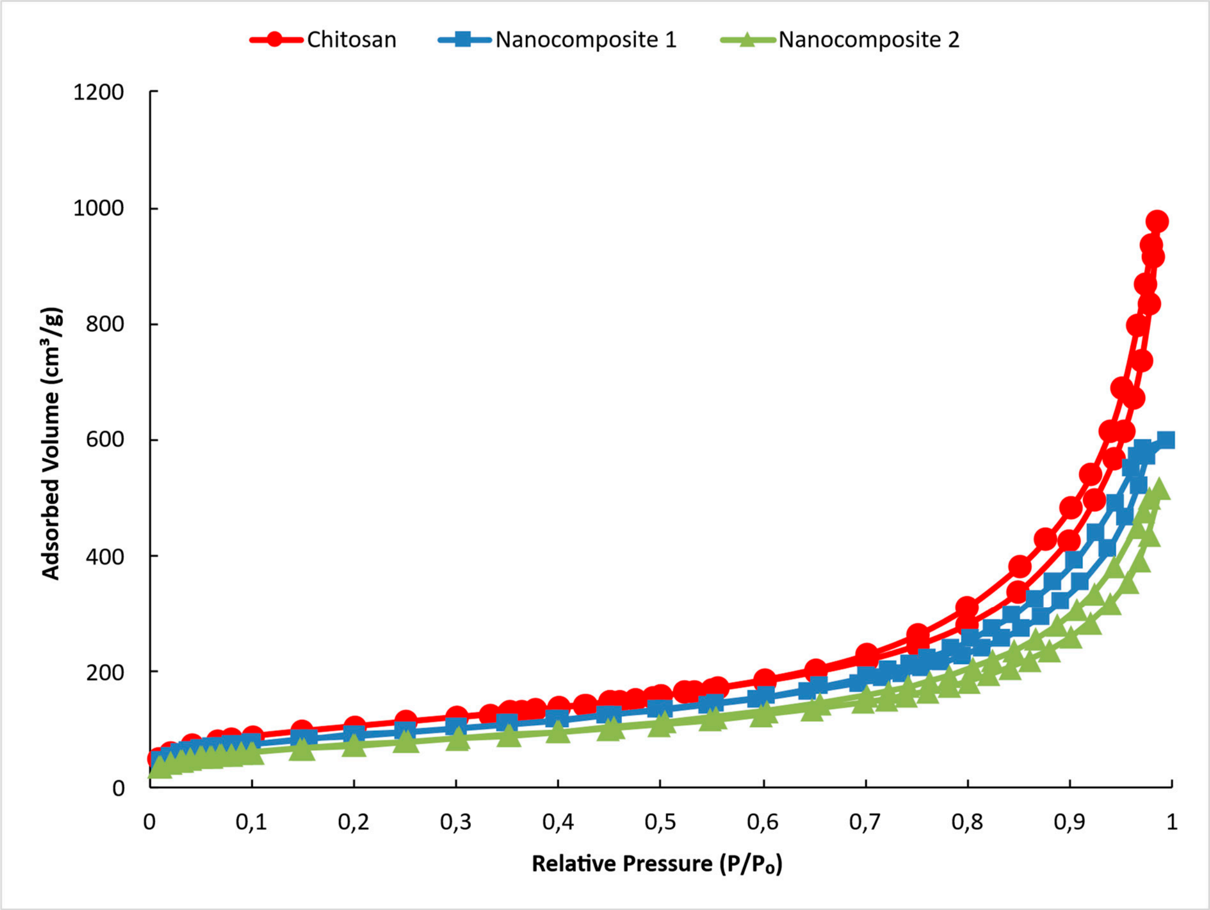

Figure S2. Nitrogen adsorption-desorption isotherms for pristine chitosan beads (-●-),$Fe_4[Fe(CN)_6]_3$/chitosan (**1**) (-■-) and $Zn_3[Fe(CN)_6]_2$/chitosan (**2**) (-▲-).

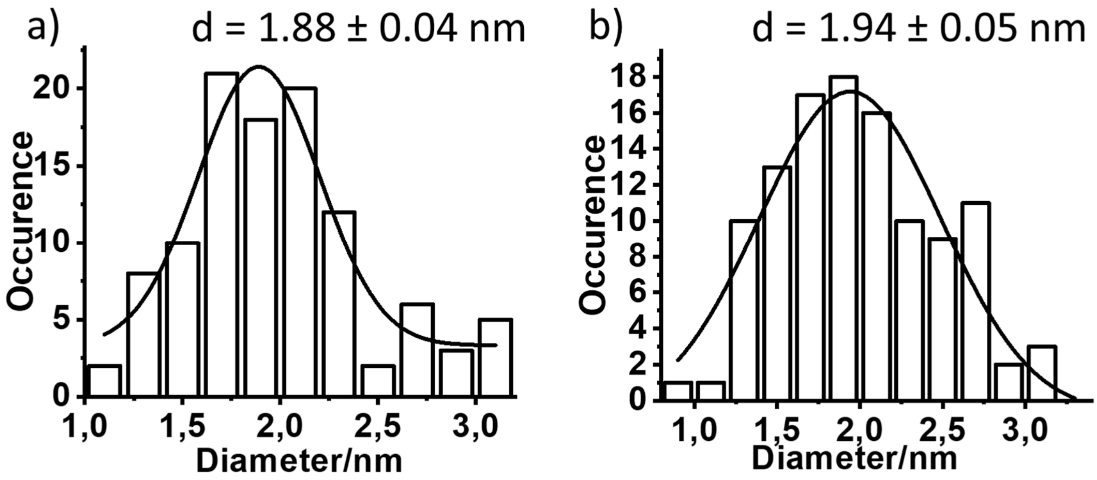

Figure S3. Size distributions of the nanoparticle size for (a) $Fe_4[Fe(CN)_6]_3$/chitosan (**1**) and (b) $Zn_3[Fe(CN)_6]_2$/chitosan (**2**).

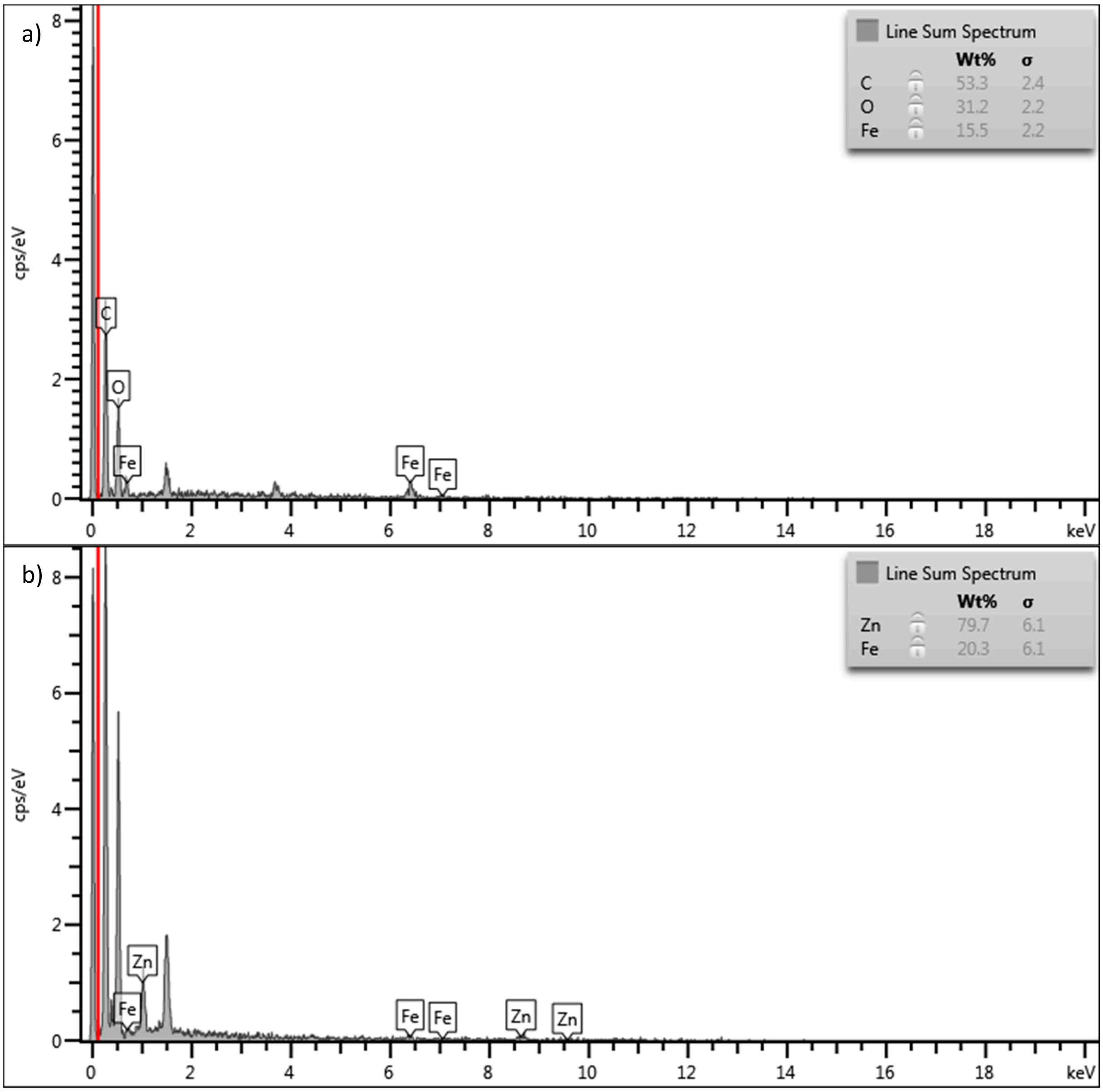

Figure S4. EDS elemental mapping for (a) $Fe_4[Fe(CN)_6]_3$/chitosan (**1**) and (b) $Zn_3[Fe(CN)_6]_2$/chitosan (**2**).
